# Epileptic spasms in *PPP1CB*-associated Noonan-like syndrome: a case report with clinical and therapeutic implications

**DOI:** 10.1186/s12883-018-1157-6

**Published:** 2018-09-20

**Authors:** Chien-Heng Lin, Wei-De Lin, I-Ching Chou, Inn-Chi Lee, Hueng-Chuen Fan, Syuan-Yu Hong

**Affiliations:** 1 Division of Pediatrics Pulmonology, China Medical University, Children’s Hospital, Taichung, Taiwan; 2 Department of Medical Research, China Medical University Hospital, Taichung, Taiwan; 3 Division of Pediatrics Neurology, China Medical University, Children’s Hospital, Taichung, Taiwan; 4 Graduate Institute of Integrated Medicine, College of Chinese Medicine, China Medical University, Taichung, Taiwan; 5 Department of Pediatrics, Chung Shan Medical University Hospital and Institute of Medicine, School of Medicine, Chung Shan Medical University, Taichung, Taiwan; 6 Department of Pediatrics, Tungs’ Taichung Metroharbor Hospital, Wuchi, 435 Taichung, Taiwan; 7 Department of Nursing, Jen-Teh Junior College of Medicine, Nursing and Management, 356 Miaoli, Taiwan; 8 Department of Pediatrics, China Medical University Hospital, 2 Yuh-Der Road, Taichung, 404 Taiwan

**Keywords:** Epileptic spasms, PPP1CB, Noonan syndrome-like disorder with loose anagen hair-2, NSLH2, Ketogenic diet, KD

## Abstract

**Background:**

Noonan syndrome-like disorder with loose anagen hair-2 (NSLH2) is an extremely rare disease caused by a heterozygous mutation in the PPP1CB gene on chromosome 2p23. The syndrome causes not only numerous dysmorphic features but also hypotonia, developmental delay, and even intellectual disability. We report the first case of NSLH2 in Asia and the 16th in the world. Moreover, the first case of PPP1CB-related infantile spasms. The clinical and therapeutic significance is outlined in this paper.

**Case presentation:**

We found a male infant presented with severe intractable epileptic spasms. Although certain clinical features of somatic dysmorphism were noted, numerous laboratory and neuroimaging studies failed to identify the cause. To determine the underlying etiology, whole-exome sequencing was conducted. We identified a de novo heterozygous mutation, NM_206876.1: c.548A > C (p.Glu183Ala), in the PPP1CB gene. His seizures were almost refractory to conventional antiepileptic drugs but relative seizure control was eventually achieved with a ketogenic diet.

**Conclusion:**

This result expands the clinical spectrum of NSLH2 and strengthens the association between the PPP1CB gene and epileptic seizures. Furthermore, we suggest that the ketogenic diet can offer seizure reduction in particular drug-resistant epilepsy syndromes. Additional studies are warranted to clarify the pathogenic mechanisms underlying this PPP1CB mutation in epileptic seizures.

## Background

PP1-beta (symbolized PPP1CB) is a catalytic subunit of protein phosphatase-1 (PP1). PP1 is one of the four major serine/threonine-specific protein phosphatases involved in the dephosphorylation of numerous proteins. These enzymes work in opposition to protein kinases, which are implicated in the regulation of cell growth, proliferation, survival, differentiation, and cytoskeletal changes, to control the level of phosphorylation [1]. PPP1CB is expressed abundantly in the human brain and spine [2, 3].

In 2014, Hamdan et al. reported a patient with a de novo 1-bp insertion in the PPP1CB gene (c.909dupA: p.Tyr450Ilefs*92), which was predicted to cause a frameshift mutation and premature termination codon. The patient’s clinical features included severe intellectual disability, short stature, relative macrocephaly, large mouth, malar hypoplasia, and mildly increased cerebral spinal fluid spaces [4]. Subsequently, Gripp et al. and Ma et al. have respectively studied four and eight unrelated patients with mutations in the PPP1CB gene. All these patients exhibited relative or absolute macrocephaly, prominent forehead, low-set and posteriorly rotated ears, and developmental delay, as well as slow-growing, sparse, or unruly hair [3, 5]. The PPP1CB gene has been associated with Noonan syndrome-like disorder with loose anagen hair syndrome (NSLH2).

Herein, we report a male infant with a PPP1CB mutation who presented not only with typical clinical features of NSLH2 but also epileptic spasms that were almost refractory to conventional antiepileptic drugs (AEDs). Eventually, relative seizure control was achieved through administering a ketogenic diet (KD). To the best of our knowledge, this is the first case of NSLH2 in Asia and the first case of PPP1CB-related infantile spasms. The clinical and therapeutic significance is outlined in this paper.

## Case presentation

The proband patient was the first child of nonconsanguineous healthy parents. His mother gave birth to him at the age of 35 years without any complication. The proband was born at 39 weeks of gestation. His birth weight was 3100 g (30th percentile), his height was 49 cm (~32nd percentile), and his head circumference (HC) was 36.2 cm (90th percentile). The Apgar score was 8/10/10 at birth. The patient did not exhibit any perinatal brain injury, hypoxia, ischemia, cranial trauma, or infection of the central nervous system. Family history of complications, such as epilepsy, febrile convulsion, intellectual disability, and psychotic disorders, was also negative.

A general physical examination at 4 months revealed macrocephaly (HC: 43.5 cm, 90th percentile), prominent forehead, low-set and posteriorly rotated ears, and sparse and slow-growing hair.

His neurological and developmental examination at 4 months demonstrated poor head control, hypotonia, less eye–object pursuit, and a less pronounced smile, but cooed occasionally. A follow-up at 14 months revealed that the patient was unable to sit stably or crawl even under an intensive early intervention.

**Fig. 1:**
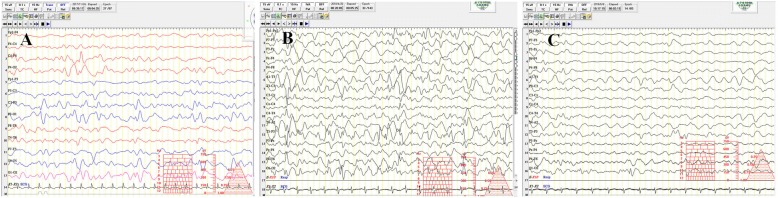
The interictal electroencephalogram at the age of 4 months consists of multifocal spikes and high-voltage wave discharges appearing mainly in the posterior region (**a**). At 9 months, the interictal electroencephalogram showed a pattern of high-voltage, chaotic, random, and multifocal spikes with or without superimposed in slow waves, and spike and wave activities in almost all cortical areas (**b**). At 13 months, (after 3 months of the KD), his electroencephalogram showed only a few spikes in the posterior region (**c**)

Paroxysmal epileptic spasms and occasional erratic myoclonic seizures in clustered episodes were first noticed at the age of 4 months, and their frequency gradually increased with age: at 9 months, the patient experienced more than 20 episodes per day, with each episode lasting 1–2 min. An initial interictal electroencephalogram (EEG) at 4 months revealed multifocal spikes and high-voltage wave discharges predominantly in the posterior region (Fig. 1a). At 9 months, the interictal EEG evolved into a pattern of chaotic, disorganized rhythms with superimposed multifocal spike discharges (Fig. 1b).

Numerous AEDs in different combinations were administered at various time points since the patient was 4 months old, namely phenobarbital (5 mg/kg/day), clonazepam (titrated to 0.1 mg/kg/day), topiramate (titrated to 9 mg/kg/day) and vitamins (pyridoxine, pyridoxal phosphate, biotin, folic acid, and vitamin B12), levetiracetam (titrated to 50 mg/kg/day), valproic acid (titrated to 30–40 mg/kg/day), and vigabatrin (titrated to 150 mg/kg/day). However, none of these treatments were effective in treating his seizures at the age of 10 months, at which time he was placed on a KD.

The following laboratory tests were conducted: urinary organic acids and an acylcarnitine profile; urine toxicology screen; blood gases and electrolytes (calculate anion gap); carbohydrate-deficient transferrin and biotinidase activity; serum chemistry of liver function tests, lactate, pyruvate, ammonia, and amino acids; cerebrospinal fluid (lactate, protein and glucose levels, cell count and microscopic differentiation, gram stain and cultures, bacterial antigens, polymerase chain reaction for herpes simplex virus and varicella-zoster virus). The results were all negative. Brain magnetic resonance imaging did not reveal any marked structural abnormalities. In addition, *cardiac sonography* at 4 months showed an atrial septal defect and peripheral pulmonary stenosis. Therefore, whole-exome sequencing (WES) was conducted to determine the underlying etiology.

Genomic DNA for WES was extracted from blood from affected and unaffected parents. Exome capture involved the use of the Agilent Human exome V5 (51 Mb) capture kit. Sequencing was performed by 101 bp paired-end sequencing on Hiseq2000 platform (Illumina, San Diego, USA) with an ~ 150× depth of coverage. Raw reads were aligned to the Human genome (hg19/GRCh37) using the Burrows-Wheeler transform (BWA-MEM; v0.7.8). Variant calling was performed using the recommended best practices of GATK version 1.0.5506 (Broad Institute). Variant annotation and prioritization were performed using a well-developed pipeline, wANNOVAR [6, 7]. Candidate pathogenic variants were defined as missense, nonsense, splice-site and frameshift mutations with a minor allele frequency lower than 0.01, using the 1000 Genomes Project, dbSNP annotations, the Exome Aggregation Consortium (ExAC) and the Genome Aggregation Database (gnomAD). Several prediction programs: PolyPhen-2, Mutation Taster, CADD, and SIFT were used [8].

**Fig. 2:**
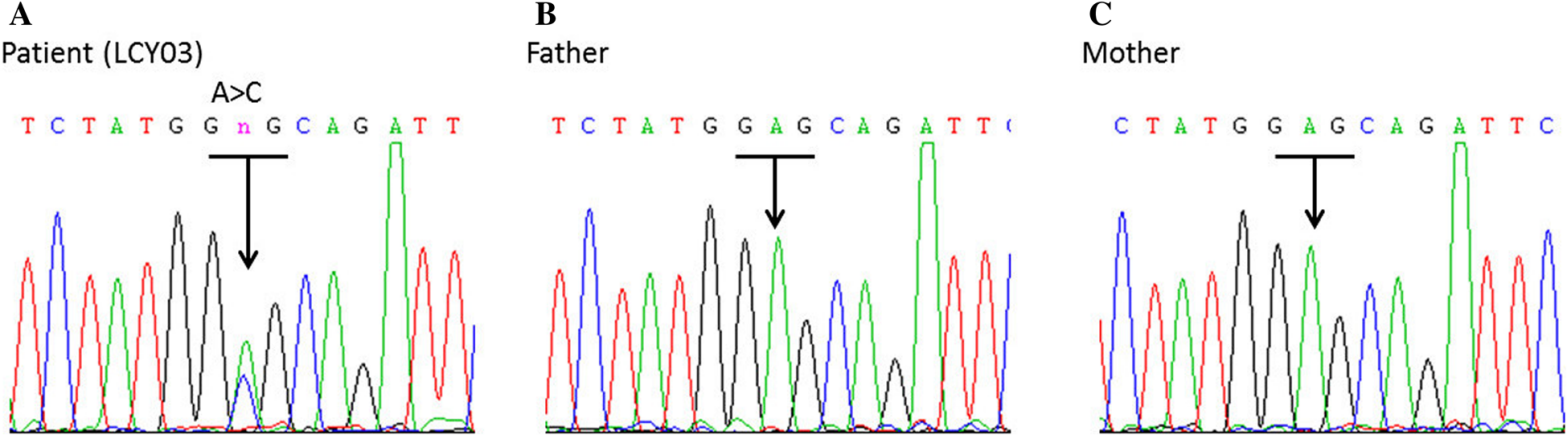
Sequencing results of the de novo mutation in the *PPP1CB* gene. The arrow indicates an A-to-C substitution at nucleotide 548 (c.548A > C, p.Glu183Ala) in the patient (**a**). This gene was normal in his father (**b**) and mother (**c**)

Only a single heterozygous missense mutation, namely NM_206876.1: c.548A > C (p.Glu183Ala), was identified in the PPP1CB gene. Variant description and *mutation* pathogenicity *prediction* were summarized in Table 1. Sanger sequencing of a DNA sample confirmed that the variant in the patient did not exist in his parents (Fig. 2). Thus, the c.548A > C variant from the proband was confirmed to be de novo. The mutation was not identified in the 1000 Genomes Project, the gnomAD, or in ExAC or dbSNP databases. Additionally, the same variant was ever reported as pathogenic by Ma et al. in 2016 [3].

**Table 1:** Method of whole-exome sequencing and the PPP1CB mutation identified in the patient

Patient ID	Variant with NM No.	AF in		*Mutation* pathogenicity *prediction*				
		1KGP	gnomAD	Polyphen HDIV score (pred)	SIFT_score (pred)	LRT score (pred)	Mutation Taster score (pred)	*Reference*
LCY03	PPP1CB: NM_206876.1: c.548A > C (p.Glu183Ala)	–	–	0.014(B)	0.01(D)	0(D)	0.81 (D)	Ma et al. [3].

*Pred* prediction, *AF* allele frequency, *1KGP* 1000 Genomes Project; *gnomAD* The Genome Aggregation Database; −, not present, *B*, benign *D* deleterious, *SIFT* sorting intolerant from tolerant, *LRT* Likelihood Ratio Test

After administering numerous AEDs in different combinations without a favorable response, we introduced a KD since the patient was 10 months of age. The diet protocol suggested by Neal et al. [9]. was followed. We started by administering medium-chain triglycerides from an infant formula and carefully monitored his blood and urine glucose and ketones, liver and renal functions, triglyceride and cholesterol levels, electrolytes, growth, and body weight gain during the treatment period. Parents, nurses, child nutritionists, and caretakers were enrolled in this treatment plan and educated regarding suitable food items, in addition to the calculation and preparation of ketogenic meals. Subsequently, the spasms gradually decreased to less than 0–2 times per day, and no cluster form was noticed at the follow-up time of 14 months of age, although seizures were occasionally exacerbated during a febrile illness episode. An EEG at 13 months revealed few spikes, mainly in the posterior region (Fig. 1c). However, developmental delay with cognitive and gross motor impairment was still observed.

## Discussion and conclusion

A de novo PPP1CB variant (c.548A > C:p.Glu183Ala) was detected through WES. To the best of our knowledge, this is the first reported case of NSLH2 in Taiwan and the 16th in the world [3, 10, 11]. Compared with other rare diseases, a PPP1CB-related disorder is a relative new disease not generally known for its phenotype, genotype, or phenotype–gene relationship.

Since the first case of PPP1CB mutation was reported in 2014,^4^ several missense mutations in *PPP1CB* associated with NSLH2 have been identified by GeneDx and numerous studies, namely c.146C > G (p.Pro49Arg), c.166G > C (p.Ala56Pro), c.548A > C (p.Glu183Ala), c.548A > T (p.Glu183Val), c.658C > T (p.Arg220Cys), c.754G > T (p.Asp252Tyr), and c.820G > A (p.Glu274Lys) [3, 5, 10, 11].

On the basis of the clinical features of NSLH2 (Phenotype MIM No. 617506) summarized in Online Mendelian Inheritance in Man (OMIM) (https://www.omim.org), its characteristics include intellectual disability, relative or absolute macrocephaly; prominent forehead; low-set and posteriorly rotated ears; congenital heart disease including atrial septal defect, mitral and tricuspid valve insufficiency, hypoplastic left-sided aortic arch, coarctation of the aorta, dilated aortic root, and peripheral pulmonic stenosis; brain abnormalities including septo-optic dysplasia and mild ventriculomegaly; connective tissue abnormalities including high-arched palate, joint hypermobility, and translucent or doughy skin consistency; and epidermal features such as slow-growing, sparse, or unruly hair. Our patient’s features exhibited a resemblance to other cases except that this patient experienced epileptic spasms; this was the first observed patient with a *PPP1CB* mutation who had epileptic spasms.

The seizure mechanism and its response to the KD remain unknown. However, Noonan syndrome or Noonan-like syndrome, diseases typified by a disturbance of the transduction signal through the RAS/mitogen-activated protein kinase (MAPK) signal pathway are related to certain genes, such as PTPN11, SOS1, RAF1, KRAS, SHOC2, BRAF, NRAS, MAP2K1, RIT1, SOS2, LZTR1, A2ML1 and PPP1CB [5, 12, 13]. Notably, although seizure disorder (13%) is not uncommon in patients with Noonan syndrome [14], neurological impairment in patients with mutations in the RAS/MAPK pathway could be more severe and linked to certain forms of refractory epilepsy, especially epileptic encephalopathy and infantile spasms [15–17].

Strategies for treating RAS/MAPK-related seizures have not been established. In the aforementioned studies, Adachi et al. [17] reported two individual cases of cardiofaciocutaneous syndrome with severe neurological impairment and infantile spasms caused by heterozygous mutations in KRAS and BRAF genes. None of the AEDs were effective in treating their seizures, but the patient with KRAS mutation (p. D153V) had a partial response to ACTH therapy. The clinical and genetic characteristics of RAS/MAPK-associated epilepsy syndromes are summarized in Table 2 [15–18]. In recent studies, the ketogenic diet has proven to be a safe and effective treatment for children with not only drug-resistant epilepsies but also epileptic encephalopathies [19–23]. However, some adverse effects that can happen in the short-term when using dietary treatment of KD should be kept in mind and closely monitored, e.g. gastrointestinal symptoms; dyslipidemia; hypoglycemia; hyperuricemia; hypoproteinemia; hypomagnesemia; hyponatremia; hepatitis and metabolic acidosis; nephrolithiasis; osteopenia, osteoporosis and bone fractures; growth failure. Fortunately, our patient further proves the efficacy of the ketogenic diet for PPP1CB-related Noonan syndrome presenting as infantile spasms and no obvious side effects were observed [24]. However, whether the KD would also have been effective in our patient for treating earlier seizure occurrences could not be investigated. Moreover, limited data for epileptic seizures associated with PPP1CB mutations are available in the worldwide population. However, more in-depth studies with additional patient data to explore the efficacy of the KD for treating epileptic seizures caused by PPP1CB mutations are essential. In conclusion, our study expands the clinical spectrum of NSLH2 and strengthens the association between the PPP1CB gene and epileptic spasms. Furthermore, we suggest that the KD be considered when seizures are *medically refractory.* Additional investigations are warranted to clarify the genotype–phenotype correlations of several genes in the RAS/MAPK signaling pathway and their associations with epileptic seizures or encephalopathies.

**Table 2:** Clinical and genetic characteristics of RAS/MAPK-associated epileptic encephalopathies

Clinical manifestation	RAS/MAPK-associated genes			
	*BRAF*	*MEK1*	*KRAS*	*PPP1CB*
Associated syndrome	CFC	CFC3	Noonan syndrome 3	NSLH2
Reported mutations	L485F; L485S; F468S; Q257R; del E11; F595 L; T599R; G534R; D638E; K499 N	F53F; Y130N	D153V	E183A
Age of onset of epileptic encephalopathies	Birth to 11y	2 reported patients (1y and15y)	3 m with Myo/11 m with IS	4 m
Seizure type	CPS, GTCS or sGTCS, Abs, IS, Tonic spasms, SE	GTCS, Abs, CPS	IS, Myo	IS, Myo
Interictal EEG	Hypsarrhythmia (some patients)	Focal epileptiform discharges to hypsarrhythmia.	Hypsarrhythmia (11 m); asynchronous slow waves with irregular spike-wave or polyspikes with and without waves (6 y).	Chaotic, high voltage, polymorphic delta and theta rhythms with superimposed multifocal spikes and wave discharges.
Development delay	Severe	Severe	Severe	Moderate to severe
Seizures refractory to AEDs or required multiple AEDs use	Yes	Yes	Yes	Yes
Seizure prognosis	Difficult to control	Difficult to control	Controlled with ACTH	Favorable to KD
Brain MRI	Cortical atrophy, hypoplastic CC	Hydrocephalus, cortical atrophy	Cortical atrophy, agenesis of the CC	Unremarkable
References	[15–18]	[15]	[18]	The present study

*CFC* Cardiofaciocutaneous syndrome, *CFC3* Cardiofaciocutaneous syndrome 3, *GTCS* generalized tonic-clonic seizure, *sGTCS* secondarily generalized tonic-clonic seizure, *CPS* complex partial seizure, *Abs* absence seizure, *Myo* myoclonic seizure, status epilepticus, *SE, IS* infantile spasms, *AEDs* antiepileptic drugs, *ACTH* adrenocorticotropic hormone, *KD* ketogenic diet, *MRI* magnetic resonance image, *CC* corpus callosum, m: months, *y* years

## Abbreviations

AEDs: antiepileptic drugs
EEG: electroencephalogram
HC: head circumference
KD: ketogenic diet
MAPK: mitogen-activated protein kinase
NSLH2: Noonan syndrome-like disorder with loose anagen hair-2
WES: whole-exome sequencing
